# Mainstreaming public involvement in a complex research collaboration: A theory‐informed evaluation

**DOI:** 10.1111/hex.13070

**Published:** 2020-05-19

**Authors:** Fiona Ward, Jennie Popay, Ana Porroche‐Escudero, Dorcas Akeju, Saiqa Ahmed, Jane Cloke, Koser Khan, Shaima Hassan, Esmaeil Khedmati‐Morasae

**Affiliations:** ^1^ Division of Health Research Faculty of Health and Medicine Lancaster University Lancaster UK; ^2^ CLAHRC North West Coast University of Liverpool Liverpool UK; ^3^ Department of Health Services Research Institute of Psychology, Health and Society University of Liverpool Liverpool UK; ^4^ Research Fellow in Policy and Complex Systems University of Exeter Exeter UK

**Keywords:** community involvement, evaluation, mainstreaming, Public Adviser, public involvement, theoretical framework

## Abstract

**Introduction:**

There is an extensive literature on public involvement (PI) in research, but this has focused primarily on experiences for researchers and public contributors and factors enabling or restricting successful involvement in specific projects. There has been less consideration of a ‘whole system’ approach to embedding PI across an organization from governance structures through to research projects.

**Objective:**

To investigate how a combination of two theoretical frameworks, one focused on mainstreaming and the other conceptualizing quality, can illuminate the embedding of positive and influential PI throughout a research organization.

**Methods:**

The study used data from the evaluation of a large UK research collaboration. Primary data were collected from 131 respondents (including Public Advisers, university, NHS and local government staff) via individual and group interviews/workshops. Secondary sources included monitoring data and internal documents.

**Findings:**

CLAHRC‐NWC made real progress in mainstreaming PI. An organizational vision and infrastructure to embed PI at all levels were created, and the number and range of opportunities increased; PI roles became more clearly defined and increasingly public contributors felt able to influence decisions. However, the aspiration to mainstream PI throughout the collaboration was not fully achieved: a lack of staff ‘buy‐in’ meant that in some areas, it was not experienced as positively or was absent.

**Conclusion:**

The two theoretical frameworks brought a novel perspective, facilitating the investigation of the quality of PI in structures and processes across the whole organization. We propose that combining these frameworks can assist the evaluation of PI research.

## 1 INTRODUCTION

Public involvement (PI) is an integral element of much health research, often required by funders. It is promoted as a means of quality improvement: increasing research relevance, and improving study participant recruitment and retention.^1^ It is also advocated on ethical and political grounds, promoting values of justice and fairness and increasing democratic accountability for public funds.^2^

Previous research, primarily focused on PI in specific projects, has highlighted the following as contributing to ‘successful’ PI: clear systems to recruit and support public contributors; targeted communication to raise public awareness about opportunities available^2^; resources to ensure appropriate recruitment^3^ and recognize the input of public contributors,^4^, ^5^, ^6^ involvement from early stages in the research process^7^; and the ability to tailor roles to both project requirements and the needs of public contributors.^8^ Training for public contributors has also been identified as important, particularly where they take on technical roles.^5^ Greater impact has been reported where goals for PI have been defined early and are reflected in implementation plans.^7^ Studies also suggest that dedicated co‐ordination and support roles in research teams facilitate the embedding of PI in projects.^3^, ^4^, ^6^

A recurrent research finding is that researchers need to build and nurture relationships with public contributors so they feel supported and part of the research team.^3^, ^5^, ^8^, ^9^ This requires researchers to have both time and a positive attitude about the value of PI.^2^ Training for researchers to address differences in knowledge and experience of PI and opportunities to reflect and share best practice have been advocated.^2^, ^3^, ^8^ More reflective practice may also enable researchers to provide feedback on the value of public contributions, facilitating their learning and development and motivating further involvement.^10^, ^11^

In addition to project‐specific barriers and enablers, there is widespread agreement that effective PI in research requires a whole‐system approach. Some studies have pointed to elements of this ‘systems’ approach suggesting, for example, that for the benefits to be maximized public contributors need to be involved throughout the research process (from priority setting to dissemination of findings) and research leaders need to be committed to/champions for PI.^2^, ^5^, ^6^, ^12^ Other factors shown to impact on the quality of PI in decision‐making in general and research in particular include opportunities for cross‐organization learning, investment in dedicated involvement infrastructure and an ‘involvement culture’.^5^, ^6^, ^8^, ^13^, ^14^, ^15^ Specific elements of organizational culture identified as contributing to successful PI include the following: organizational commitment to learning and changing in response; non‐hierarchical collaboration between professionals and public contributors; and staff behaviour reflecting mutual recognition and respect.^9^

Notwithstanding these important insights, knowledge about how a ‘whole system’ approach to embedding and sustaining high‐quality PI across research organizations is patchy and potentially important areas, such as PI in organizational governance, are particularly neglected. This paper aims to contribute evidence in this area by presenting findings from an evaluation of the approach taken by a large research collaboration to embedding PI across all organizational levels, processes and activities. Two complementary theoretical frameworks informed the study: an established mainstreaming framework^16^ with a strong organizational focus and the Involvement Cube^17^ focusing on aspects of the quality of PI. The rationale for selecting these is discussed further below.

## 2 STUDY SETTING AND DESIGN

The evaluation was based in the English Collaboration for Leadership in Applied Health Research and Care for the North‐West Coast (CLAHRC‐NWC). The National Institute for Health Research (NIHR) funded thirteen CLAHRCs in two waves from 2008 to 2013 and from 2014 to 2019. Each CLAHRC comprised a network of partner organizations from the public, private and not‐for‐profit sectors and aimed to support capacity building for the conduct and translation of applied health research to improve population health and reduce health inequalities.

CLAHRCs were expected to involve the public in their work.^18^ However, evaluation of their experience is limited. In the first wave CLAHRCs, only three projects investigated how PI was ‘enacted’ and how roles developed as part of wider evaluations^9^, ^12^, ^19^, ^20^ whilst a more recent study used an action research approach to look explicitly at enablers and barriers to PI in research in a CLAHRC.^8^

CLAHRC‐NWC was established in 2014 and had 36 formal partners (Universities, NHS Organizations, Local Government and Industry) working alongside members of the public and not‐for‐profit organizations. It undertook an internal evaluation during 2017/2018, which included a focus on its approach to PI. The evaluation focused on four components: the Neighbourhood Resilience research programme (NR) addressing local social determinants of health inequities; the Partner Priority Programme's action research on new models of care (PPP); the Intern programme (IP) providing research training for NHS and local government staff; and the extent to which strategic objectives for PI, health equity and research capacity building were achieved across the collaboration (CC).

The evaluation was primarily qualitative to enable in‐depth investigation^21^ with content analysis of internal documents and analysis of routine monitoring data undertaken to varying degrees across the four components. It was conducted by teams of academics and Public Advisers. In addition, a panel of six Public Advisers contributed to the study design and the interpretation and dissemination of findings. Importantly, the academics leading and conducting the evaluation had research roles in CLAHRC‐NWC and no responsibility for strategic delivery of, or support for, PI.

In total, data were obtained from 131 individuals: semi‐structured face‐to‐face interviews (n = 58) and group interviews/workshops (n = 73). These included staff from CLAHRC‐NWC's NHS, local government, university and not‐for‐profit partners; Public Advisers; and professional interns (Appendix S1). Information sheets and consent forms emphasized that participation was voluntary. Ethical approval was obtained from the university where the lead researchers were based: Lancaster University for research on the Neighbourhood Resilience programme and CLAHRC‐NWC strategic objectives (FHMREC13028, FHMREC17023); University of Liverpool for the Partners Priority Programme research (2236); and University of Central Lancashire for research on the Intern programme (STEMH608).

The findings reported here are based on qualitative data exploring processes, experience and impact of PI from all four evaluation components, key documents and PI monitoring data. As each evaluation component had its own objectives, interview topic guides varied: those used to investigate CLAHRC‐NWC's strategic objectives contained detailed prompts about PI; those for the intern interviews focused on whether new collaborations had been formed and who helped with the conduct of the research; the PPP group interviews asked about their experience of undertaking research within CLAHRC‐NWC and things they would do differently as a result. Interviews for the neighbourhood programme evaluation focused on involvement of residents and asked if their experience had impacted on other activities they were involved in. The volume and detail of data therefore varied across components but together provided a ‘thick’ picture of PI across CLAHRC‐NWC.

All interviews were recorded and transcribed. A combination of deductive and inductive approaches was used to develop an coding frame, based initially on existing theory around mainstreaming and PI, as the analysis evolved additional codes were added.^22^ Researchers familiarized themselves with the data by reading the transcripts, noting new codes. The final coding frame was uploaded to Excel, systematically applied to all transcripts with the first 20 tested by two researchers. CLAHRC‐NWC policies and strategies and Steering Board minutes were reviewed to identify references to PI, and routine PI monitoring data were utilized. Data were coded into a set of analytical charts, which were studied to identify common or divergent perspectives and discussions within the team identified potential interpretations. The Adviser panel participated in two workshops discussing preliminary findings. Quotations used to illustrate findings are referenced by data collection method, respondent number, organization and evaluation component (eg int14‐NHS‐CC is interviewee 14, an NHS Partner in the Cross‐CLAHRC evaluation).

## 3 THEORETICAL FRAMEWORKS

**FIGURE 1:**
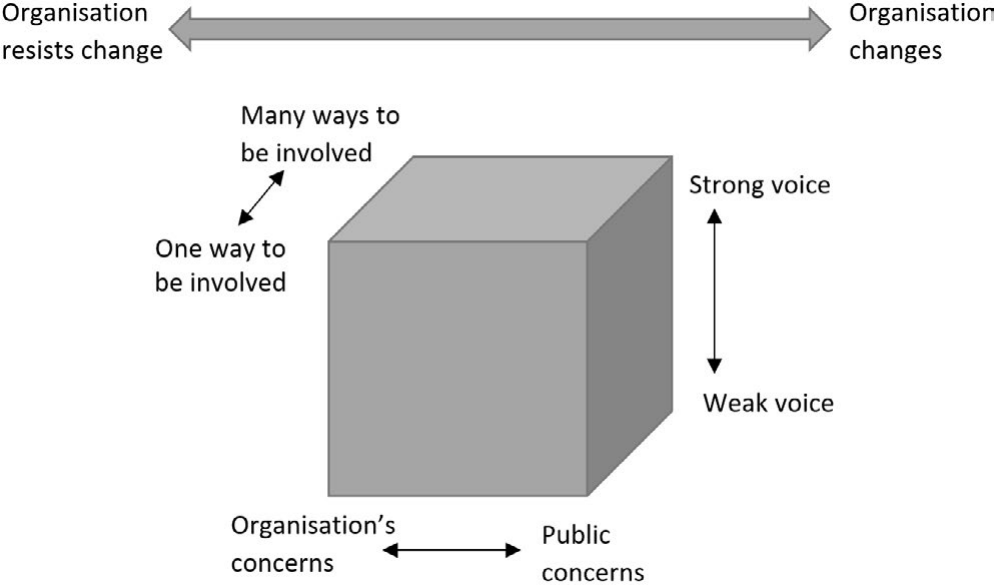
Involvement cube (as revised by Gibson et al)^21^

CLAHRC‐NWCs aspirations for PI had two dimensions. The first was to incorporate ‘the public, including patients, service users and carers and members of communities of interest and place across all of our work.’ In effect, PI was to be mainstreamed across the organization, that is ‘accepted as normal by most people’.^23^ To assess progress towards meeting, this aim we drew on the gender mainstreaming framework developed by Moser and Moser.^16^ This identifies three organizational domains in which change is required for successful mainstreaming: culture; policy development; and policy implementation. Exploration within and across these domains enables ‘a synthesis of progress and the identification of limitations and gaps’.^16^ Secondly, CLAHRC‐NWC aimed to ensure that the public had influence across the collaboration and PI would be a positive experience. To examine achievements in these domains, we drew on the Involvement Cube^17^, ^24^ (Figure 1) which identifies four dimensions of ‘quality’: (a) the extent to which different forms of knowledge and understanding are valued; (b) the ‘strength’ of the public voice; (c) the diversity of involvement approaches; and (d) professional and organizational commitment to listen and act on the ‘publics’ priorities.

In selecting these two theoretical frameworks, we were mindful of Edelman and Barron's argument that evaluating PI as if it were a complex intervention can result in too narrow a focus.^25^ The diversity of theoretical and methodological approaches used in evaluations of PI in research has helped address this risk. However, two alternative approaches most relevant for our study are underpinned by an intervention perspective: Normalization Process Theory^26^ developed to assess how complex social interventions are embedded and sustained in practice and Realist Evaluation^27^ developed to assess whether ‘a policy works, for whom, in what circumstances'. In contrast, the gender mainstreaming framework has an explicit focus on organizational cultural and structural change. We wished to test the added value of combining this framework with one focused on drivers of quality PI.

## 4 FINDINGS

### 4.1 Progress on mainstreaming public involvement

#### 4.1.1 Cultural change: adopting the terminology and sharing a vision

The first stage of the mainstreaming process requires the development of shared understandings about PI to be articulated and communicated across an organization. CLAHRC‐NWC's vision to ‘…involve patients and the public in decision making at all levels’ was elaborated in the original funding proposal.^28^ Once CLAHRC‐NWC was established in 2014, the terminology was developed further in a Strategy for Stakeholder Engagement and a Policy for Public Engagement. These re‐stated the vision, principles and objectives and the ‘diverse’ routes for PI including the Steering Board and Management Team; a Public Reference Panel; Theme Management; research and knowledge mobilization projects; and capacity building activities.

From the start, a university professor as Director of Public and Partner Engagement signalled the importance of PI. Two public contributors were involved in developing the funding bid and sat on the Management Team. A senior manager suggested they were ‘active and instrumental’, strongly influencing the thinking about PI and the language used. Having an articulated vision at this early stage was felt to have communicated an organizational commitment and created expectations about PI. As one Partner noted: ‘I think that's been really important, setting your stall out right away saying this is what you have to do’ (int14‐NHS‐CC).

Importantly, PI terminology evolved over time. Initial documentation reveals deliberations about the meaning of, and distinction between, ‘engagement’ and ‘involvement’. Over time ‘involvement’ was used more consistently to reflect the aspiration for all the collaboration's activities to be conducted ‘with’ or 'by’ members of the public. The early title of ‘public volunteer’ was also discarded in favour of ‘Public Adviser’ to give more formal status to their role, and the Public Reference Panel was renamed the Adviser Forum to ‘signal that it was an inclusive space for all Public Advisers’ (int16‐University‐CC).

#### 4.1.2 Putting the policy in place—organizational processes and infrastructure for involvement

**FIGURE 2:**
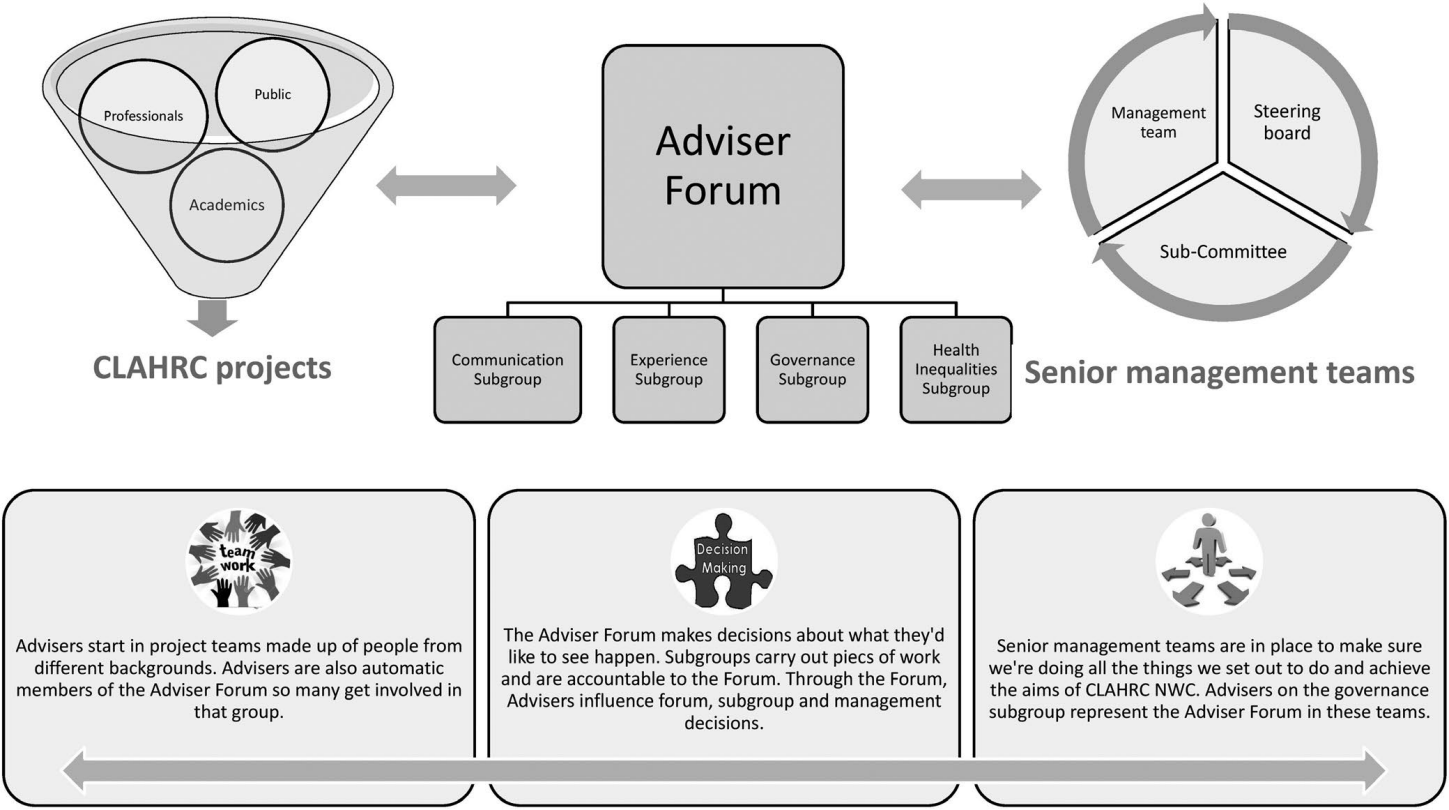
Public involvement in CLAHRC‐NWC

Two public contributors took a leading role in producing the initial policies to support and promote PI throughout the collaboration. These included policies on reimbursements, training, personal development, communication and monitoring. The PI infrastructure was established by November 2014: comprising the Director of Engagement, a full‐time facilitator and a little later, a part‐time assistant and dedicated budget. Processes were put in place for the recruitment of Public Advisers and the payment of fees/expenses. Registered Advisers automatically became members of the newly created Public Reference Panel/Adviser Forum, which was to oversee implementation of the PI policy and send representatives to the Steering Board, its funding sub‐committee and the CLAHRC‐NWC Management Team. Thus, as illustrated in Figure 2, through membership of the Panel/Forum, the public voice was present across the collaboration's central governance structures.

The infrastructure also developed over time. For example, a senior manager joined the central team to support the involvement of residents of disadvantaged neighbourhoods and the Adviser Forum established four subgroups to help it work more effectively and support Advisers to develop specialist knowledge.

#### 4.1.3 Implementation: public involvement in practice

For PI to be mainstreamed, we would expect to find evidence of involvement in all aspects of CLAHRC‐NWC's work and positive attitudes towards, and increasing confidence about, PI amongst professionals.

By in 2018‐2019, there were 170 registered Public Advisers and they contributed almost 20:00 hours to research, governance and training. Advisers felt strongly that they were a valuable resource for CLAHRC‐NWC and as their numbers grew, they brought an increasingly wide range of skills and multi‐faceted experiences of health conditions, as service users, carers and residents of disadvantaged neighbourhoods:

If you're the service user you're just as much of a professor as they are because you been through the system for 20 years, 30 years; you've got more experience. So that's what you've got to try and bring to it. (grp3‐Adviser‐CC)

There was widespread recognition that the timing of PI was critical to the benefits delivered. Respondents commented that early involvement meant the public could influence critical decisions about the focus and design of projects, ensuring the work was relevant and that professionals avoided ‘…falling into a trap of unfortunately thinking we know best, and I know we don't.’ (int34‐LocalGov‐CC). Advisers with strategic governance roles reported growing insight into the workings of CLAHRC‐NWC and confidence they were able to bring the views of the wider Adviser base to the discussions. They highlighted areas in which they felt they had an impact, including the Steering Board sub‐committee responsible for agreeing project funding, a view reinforced by this University Partner:

It's not a rubber stamping this sub‐committee… and I know that we get quite a rough time sometimes and they do give projects back and they ask for more information and they ask for more public engagement. They do point out gaps and sometimes things have been round more than once before they get through to the Steering Board. (int1‐ University‐CC)

Public Advisers also became more involved in governance roles, including in the Adviser Forum subgroups, and in the management of programmes, participating in, for example, development days and strategy groups. One University Partner explained that Advisers, staff and Partners had developed together:

It's changed hugely actually, and I think it's been partly with their confidence and CLAHRC understanding and accepting it. (int15‐University‐CC)

Over time, involvement opportunities increased with new and varied projects and a wider range of research activities including proposal development, data collection, analysis and dissemination via papers and presentations. Some Advisers said they felt they were integral members of research teams and several Partner staff felt approaches to PI had changed for the better:

…before it was very much right, we've developed a project let's go out to consultation, we will have a group of the public, we will consult with them and then we will take that information back and work on it. So, the PI is over and done in one meeting maybe. Whereas now it's sort of more integrated and they're starting to ask: what would you like to do on this project, what can you do, what would you like some support with, to help you to do this. (grp16‐University‐PPP)

Another senior academic reiterated this view, suggesting PI was ‘…much more embedded I think in a wider group of academics and researchers; so that's a really positive thing’. (int9‐University‐CC). Many partners expressed an eagerness to progress PI in their research projects as this NHS‐based respondent illustrates:

…she [PA] just asks questions, ‘well have you thought about it this way and why are you doing it that way’ and I'm like, ‘well we don't know’ and that's just somebody with an outside perspective but who is a patient. (grp16‐NHS‐CC)

Reflecting improvements over time one Adviser contrasted their current positive experience with an earlier experience of being:

…parachuted in half‐way through when you've got all the data…It's so frustrating when you've entered that thing four months down the line – you were there just to read a few proofs and you couldn't even do nothing about it anyway. (grp3‐Adviser‐CC)

However, routine monitoring data revealed that four years in, PI was still not equally embedded throughout CLAHRC‐NWC. Some project leads argued that the time required to recruit and support Advisers could be prohibitive, whilst other respondents felt where there was limited PI it largely reflected a lack of commitment by some senior staff rather than organizational barriers:

Do people really believe that this [PI] will improve the quality and relevance of their research? My personal view is that, though they wouldn't admit it, they don't because if they did they would be doing it better. (int16‐University‐CC)

Additionally, it was suggested that CLAHRC‐NWC's requirements were the only reason some projects had recruited Public Advisers, leading to tokenistic practices:

It was so frustrating to know that all the work had been done and then to have to be really civil and polite. I think they just put you there then because you tick the box. (grp2‐Adviser‐CC)

### 4.2 The quality of involvement: experience and influence

Alongside mainstreaming PI across the organization, CLAHRC‐NWC aspired to deliver involvement that was experienced as positive and influential. The findings point to progress as well as continuing challenge within the ‘quality’ domains of the Involvement Cube.

#### 4.2.1 Valuing diverse knowledges and innovating involvement approaches

Staff across partner organizations reported a growing understanding of the value that Public Advisers could bring to their projects. One local government lead described his project as a ‘…team effort with me learning as I've gone along what different people can contribute’. He highlighted how cross‐organizational structures had opened up space for innovation:

CLAHRC having an infrastructure around this does make you aware that you can do a job description for a Public Adviser and really think about the different roles they would take at different points in a project. (int3‐LocalGov‐CC)

Many Advisers recognized a positive change in the organizational culture, describing professionals across CLAHRC‐NWC as people who ‘get public and patient involvement’ (grp4‐Adviser‐CC). Another noted that when Advisers understood what was expected of them and were valued for their contribution, they could be inspired to get more involved:

We work as a team with other members of the group. That's important, that we all complement one another rather than just accepting the fact that we are there. (grp4‐Adviser‐CC)

In contrast, other Advisers did not feel listened to, or were unsure how useful their input had been. One suggested their project had been dominated by the concerns of academics ‘…assuming that the PI would follow, and they would agree with what academics decided’ (grp7‐Adviser‐CC). Another concluded that staff were not fully utilizing the public's expertise:

I think there's an education as much for the involving institution as there is for the Adviser you know about what's expected… I currently feel I've got more to offer but it's a question of how much they want to engage with me. (grp14‐Adviser‐PPP)

Despite the requirement, project teams did not always produce role descriptions or induction programmes for their Advisers, leaving them uncertain:

…it was quite welcoming. I was I think a little slow in understanding what the Public Adviser brought to the project and in some ways, I would have liked them to have been clearer about what my involvement was. (grp15‐Adviser‐PPP)

#### 4.2.2 Strengthening the public voice through support and communication

Several respondents stressed the value of the central dedicated PI posts:

…having a specialist post dedicated to supporting them and helping them to develop and encourage them; I think that's a really important part of it. (int9‐LocalGov‐CC)

These staff facilitated introductions, processed expenses and fee forms and provided logistical support for the Adviser Forum. For those Advisers involved in strategic governance, they played a significant role:

…she's the buffer between CLAHRC and us as a group and when I say a buffer it's not that we don't understand what they say at meetings it's just she is there explaining all the time what's what and do it this way, do it the right way. (grp5‐Adviser‐CC)

Clarity of language and explanations of specialist terminology facilitated understanding and enabled Advisers to contribute more fully. Similarly, clear and timely communication helped Advisers to feel ‘appreciated’. (grp7‐Adviser‐CC). Some professional respondents were also aware of the importance of good communication:

…we have been able to get more involvement of public members and that has been beneficial. It's been challenging but it's been beneficial as well. Challenging because of understanding the level that they are coming from, to adjust what we do and say to make sure that it's relevant and helpful to those individuals as well. (int8‐University‐CC)

However, language was sometimes experienced as excluding. One Adviser suggested that researchers should ‘…tone down the academic side, it frightens ordinary members’ (grp8‐Adviser‐NR) whilst another stressed that ‘communication is key and getting the messages right is key and I think sometimes the jargon that's used makes it really difficult’. (grp11‐University‐PPP). Additionally, poor communication and insufficient information had left some Advisers feeling their voice was weak and out of touch with their research project:

Nothing has happened in the past 12 weeks, well not that I'm aware of. This is another frustration ‐ it would be rewarding if they could drop an email and say well, we realise we've got you on hold you know, could you just be aware of this this and this. (grp14‐Adviser‐PPP)

#### 4.2.3 Strengthening the public voice by building skills and knowledge

There was a widespread view that members of the public needed access to development opportunities so that they could contribute fully to their chosen activities. As one Adviser highlighted:

[Name] told us as an advisory panel we are going to present, so it's given us some ideas how to present. We prepared a PowerPoint presentation, so it's given me as an Adviser some confidence to present my work, to disseminate our work as a team in front of all the [project] participants. (grp15‐Adviser‐PPP)

For some, skills development, combined with growing confidence had strengthened the Advisers' voice, enabling them to become more involved in project activities:

One Adviser was saying that they wanted to see all the Adviser comments from [project] …I think that person wouldn't have made that comment a few years ago. I don't think they would have had the confidence, but I don't think they would necessarily have had the skills to be able to evaluate the Advisers' input. (int2‐NHS‐CC)

Skills development for professionals was also important. Advisers, for example, contributed to training on construction of role descriptions which, as one university‐based respondent suggested, increased researchers' understanding of ‘added value and the skills’ (grp16‐University‐CC) that Public Advisers could bring.

#### 4.2.4 The Adviser Forum—collective involvement and influence

Advisers' experience of participation in the Adviser Forum and its subgroups demonstrated the extent and influence of PI in the strategic governance of CLAHRC‐NWC. As one Adviser noted:

The Public Reference Panel [Adviser Forum] was actually developed by the public so we were part of discussing the fees, involved in the protocols and plans, the actual welcome packs; we were part of everything. (grp3‐Adviser‐CC)

Through the Forum, supported by the central PI team, Public Advisers monitored the implementation of the Public Engagement Policy, sent representatives to the Steering Board and Management Team and responded to requests for advice. Advisers were confident that they shaped the continuing development of PI across the collaboration and there was evidence to support these views. When Advisers raised concerns about the lack of PI in some research projects, for example, their proposals to ensure the Steering Board received more transparent information were adopted. Similarly, the Forum's work on the Code of Conduct for PI led to a requirement for Public Adviser role descriptions to accompany all project proposals going to the Steering Board's sub‐committee for approval.

A collective sense of being listened to extended to Advisers choosing not to attend the Forum who suggested they had influence through fellow Advisers acting on their behalf:

…we think they [CLAHRC‐NWC] have been successful because it makes you feel a part of it and I say having these steering groups and advisory groups and sub‐groups are a big help because even if you're not involved in them, you know service users are involved. (grp2‐Adviser‐CC)

Advisers who were not members of the Forum's subgroups also felt involved and well informed about this work through peer support:

They are really good at supporting each other. A couple of people in the group have been proactive and telling us about events… I know two of the Advisers and am friendly with them, they support me and explain anything I am not sure of. (grp8‐Adviser‐PH)

## 5 DISCUSSION

The research reported here aimed to explore the extent to which positive and influential PI had been embedded throughout a large research collaboration. Some potential limitations of the study should be noted. The research was conducted by internal teams but several steps were taken to reduce potential bias^29^: Only Popay was involved in CLAHRC‐NWC governance or had any responsibility for implementing PI policy. She provided methodological advice and contributed to the interpretative process but was not involved in conduct of the research or data analysis. Additionally, team members did not interview people they had previously worked with; the initial coding frame was based on existing theory, which also informed the analysis; data extraction from a subset of transcripts was undertaken by two researchers; and the credibility of preliminary findings was tested through collective reflection within the team and during interpretive workshops with the Public Adviser panel. The nature of the data on PI varied across the four components of the evaluation but combining them provided an element of triangulation.

Our findings suggest that CLAHRC‐NWC had made substantive progress in mainstreaming PI: it was evident at all levels of the organization and many activities. There was clear evidence of the development of an organizational culture encompassing a positive vision and discourse about the place and value of PI in the collaboration. For many respondents, four years after it began, CLAHRC‐NWC was a place where people ‘got’ PI. Many, public and professionals alike, reflected how, over time, they had become more aware of the benefits and more willing to innovate in approaches to involvement.

Within a few months of CLAHRC‐NWC's launch, policies setting out structures and processes to practically embed PI across the collaboration had been co‐produced by Public Advisers and professional staff and agreed by the Steering Board. Implementation followed quickly with an infrastructure established, including a central team with a dedicated budget supporting recruitment, payment of fees, and training and development for Advisers and staff. The number and diversity of involvement opportunities grew and by CLAHRC‐NWC's final year, almost 200 Advisers were registered and contributing significant time within the collaboration.

More varied opportunities enabled Advisers from diverse backgrounds to become involved in ways that suited their interests and motivations. The developing capabilities of individual Advisers encouraged further involvement and gave them confidence to make their voice heard. There was clear evidence that the organization both listened to and acted on the priorities identified by Public Advisers. In particular, the Adviser Forum operated as a vehicle through which the public voice reached throughout the CLAHRC‐NWC.

The collaboration, however, had not fully achieved their aspirations. In some themes and projects, there was limited PI and at times the experience was less positive. A key factor here was the lack of commitment from some senior staff, communicated to their colleagues, that time invested in PI was time well‐spent.

The evaluation adopted a novel theoretical approach, combining two frameworks, one focused on mainstreaming (not used previously in this field) and the other on quality. It moved away from the project‐based design prominent in the field and sought to represent perspectives from different ‘constituent’ groups across the organization. In this way, we hoped to contribute to the more reflective approach to evaluating PI in research that Boivin has recently argued will help produce a more ‘nuanced’ understanding of how benefits can be maximized and potential risks avoided.^30^

Informed by the two frameworks, the findings illuminate the dynamic nature of PI throughout a research organization and the factors driving change at individual, project, programme and organizational levels. The mainstreaming framework focused the analysis on progress and challenges in three key organizational domains in which change was needed if PI was to become embedded across the collaboration. They also reveal that evidence of policy implementation does not necessarily mean that involvement is experienced as positive and influential by members of the public or staff. The Involvement Cube provided a theoretical lens through which to assess these ‘quality’ dimensions in terms of public and professional knowledge exchange; diversity of involvement opportunities; strength of the public voice; and the collaboration's willingness to act on the public's input.

## 6 CONCLUSION

Examining the evolution of institutional culture alongside policies, infrastructure and procedures has not been a feature of much previous PI research which has often focused at the project level, sometimes looking outwards towards organizational processes that enable PI in projects but not at the vision, structures and processes of the organization as a whole. As Moser and Moser contend, having a clear institutional stance is vital for mainstreaming success, contributing to ‘…a long‐term transformation process for the organization in terms of attitudes, ‘culture’, goals and procedures’.^16^ Our findings suggest that these elements are all critical to making progress in embedding PI across research organizations but as an evaluative tool, this framework alone is not enough. Combining it with the Involvement Cube enabled a more nuanced and quality‐focused examination of PI across the CLAHRC‐NWC, suggesting it is a useful combination of theoretical approaches to apply to future PI research.

## CONFLICT OF INTERESTS

The author(s) declared no potential conflicts of interest with respect to the research, authorship and/or publication of this article.

## Supporting information

Appendix S1

## Data Availability

Due to confidentiality, and the nature of the consent obtained, the qualitative interview transcripts cannot be shared. For further information related to this data set, please contact the corresponding author.
