# Supplementary material for: Mainstreaming public involvement in a complex research collaboration: A theory‐informed evaluation
Source: Health Expect. 2020 May 19;23(4):910–8. doi: 10.1111/hex.13070 (PMC7495077; doi:10.1111/hex.13070)
Supplement: Supplementary file 1 — Appendix S1 [file HEX-23-910-s001.docx]

Mainstreaming public involvement

**APPENDIX S1**

**Methods and samples for each dataset and topics covered**

| **Evaluation component** | **Date** | **Methods and participants** | **Topics covered** |
| --- | --- | --- | --- |
| Cross CLAHRC-NWC (CC) | Dec 2017-April 2018 | Interviews with Steering Board Chair, senior management, partners involved in research and implementation projects (n=20)  Focus group with research managers from university partners (n=7)  Participatory workshops with Public Advisers (n=26) | Capacity Building  Health Inequalities  Public Involvement |
| Capacity Building -Intern Programme (IP) | Nov 2017 | Interviews with Interns from partner organisations (n=8) | Application process  Supervision and support  Training and skills development  New networks and collaborations  Health inequalities  Outputs and implementation |
| Partner Priority Programme (PPP) | Sept 2017 – Feb 2018 | Focus groups with:  Public Advisers (n=5)  Project leads from NHS and LA partners (n=6)  Research and development leads from NHS partners (n=4)  Interns from NHS and LA partners (n=5)  University partners who developed and supported the delivery of the PPP (n=8)  Research and implementation project designers from university, local government and NHS partners (n=6) | Programme processes  Personal and organisational learning from programme involvement  Impact of involvement |
| Public Health  Neighbourhood Resilience (NR) Programme | Sept 2017 - Oct 2017 | Interviews with staff from local government partners (n=9)  Peer to Peer interviews with Public Advisers (n=21)  Focus group with community research network facilitators (n=6) | Understanding of the programme  Getting involved  Programme governance  Impact of involvement  Health inequalities |
